# SHARP-AODV: An Intelligent Adaptive Routing Protocol for Highly Mobile Autonomous Aerial Vehicle (AAV) Networks

**DOI:** 10.3390/s25247522

**Published:** 2025-12-11

**Authors:** Nguyen Duc Tu, Ammar Muthanna, Abdukodir Khakimov, Irina Kochetkova, Konstantin Samouylov, Abdelhamied A. Ateya, Andrey Koucheryavy

**Affiliations:** 1 Department of Telecommunication Networks and Data Transmission, The Bonch-Bruevich Saint-Petersburg State University of Telecommunications, Saint Petersburg 193232, Russia; nguyentuhd99@gmail.com (N.D.T.); akouch@mail.ru (A.K.); 2 Institute of Computer Science and Telecommunications, Peoples’ Friendship University of Russia (RUDN University), Moscow 117198, Russia; muthanna.asa@sut.ru (A.M.); khakimov-aa@rudn.ru (A.K.); kochetkova-ia@rudn.ru (I.K.); samuylov-ke@rudn.ru (K.S.); 3 EIAS Data Science Lab, College of Computer and Information Sciences, Prince Sultan University, Riyadh 11586, Saudi Arabia

**Keywords:** SHARP-AODV, Autonomous Aerial Vehicles (AAVs), adaptive routing, ad hoc networks, 6G, low latency, edge computing, Metaverse, Quality of Service (QoS)

## Abstract

In ad hoc networks employing Autonomous Aerial Vehicles (AAVs), the importance of real-time applications and edge computing is steadily increasing. However, existing routing protocols still fail to meet the strict performance requirements under the unique conditions of AAV networks, where the network topology changes continuously, and nodes move at high speed. This paper presents SHARP-AODV (Stability Heuristic Adaptive Routing Protocol—AODV), an enhanced routing protocol specifically developed for AAV networks. SHARP-AODV introduces two key innovations: (1) an intelligent RREQ (Route Request) dissemination mechanism that combines neighbor density control with a multi-parameter probabilistic model, and (2) a multi-criteria path selection mechanism that jointly considers hop count, link quality, and resource state. Simulation results in NS-3 across four distinct mobility models and various numbers of AAV nodes show that SHARP-AODV significantly outperforms standard AODV, improving packet delivery ratio (PDR) by up to 23.9%, increasing throughput by up to 61%, while reducing end-to-end delay by up to 87.8% and jitter by up to 90.6%. The proposed protocol is especially suitable for AAV-enabled applications in Edge Computing and Metaverse ecosystems that require low-latency, highly reliable connectivity with adaptation to dynamic network conditions. Furthermore, SHARP-AODV satisfies 6G network requirements for connection reliability, ultra-low latency, and high device density, unlocking new opportunities for employing AAVs in smart cities, environmental monitoring, and distributed VR/AR systems.

## 1. Introduction

The rapid advancement in autonomous aerial vehicle (AAV) technology has unveiled a broad spectrum of functional uses in numerous important fields of socioeconomic life. In disaster response and emergency management, AAVs can serve as important temporary mobile transceivers that rapidly restore connectivity in hurricane-flood-earthquake-isolated regions and simultaneously provide aerial reconnaissance assistance to search for casualties and assess damage in real-time [1,2,3]. In precision agriculture, AAV fleets equipped with multispectral sensors undertake comprehensive monitoring of crop health, enable prompt disease diagnosis, and improve irrigation and fertilization management with high-resolution spatial data analysis to enhance productivity with reduced environmental impacts. For environmental monitoring and conservation, AAVs facilitate continuous wide-area ecological data collection without causing disturbance, ranging from wildlife tracking and forest condition assessment to air and water quality monitoring in regions where ground-based vehicles cannot access [4,5].

In logistics and autonomous delivery, e-commerce companies and healthcare services are deploying AAVs for short-range cargo transportation, particularly express delivery, medical sample transport, and pharmaceutical distribution to remote areas, reducing delivery times from hours to mere minutes [6,7]. Moreover, within smart city contexts, AAVs integrate into intelligent transportation systems (ITS) to monitor real-time traffic flow, detect accidents, support traffic law enforcement, and even serve as platforms for future autonomous air taxi services. Finally, in entertainment and media, synchronized AAV formations create spectacular three-dimensional light shows, while AAVs equipped with high-quality cameras provide unique aerial perspectives for film production, live sports broadcasting, and even Metaverse events where AAVs function as mobile “eyes” for remotely participating virtual users [8,9,10,11]. But only through the full exploitation of this is this potential possible if reliable, low-latency network connections between AAVs and ground infrastructure can be guaranteed in the presence of high mobility and dynamic topology change, precisely the challenge SHARP-AODV was designed to address.

According to estimates by the ITU-T Focus Group on Technologies for Network 2030, AAVs will be employed as mobile base stations and relay nodes in the multi-tier architecture of 6G. Because they deploy quickly and are highly adaptable, AAVs can be deployed to cover distant or disaster-stricken areas while alleviating over-congestion in busy locations. Table 1 shows the expanding scope of AAV applications across different generations of mobile networks, the massive leap in task complexity and technology demands for the 6G era.

Despite their promise, multi-tier AAV networks face significant challenges in communications and routing stemming from the unique characteristics of these systems.

(1) High mobility in three-dimensional space

AAVs move at high speeds along altitude, longitude, and latitude, producing a rapidly varying network topology. This leads to a substantially higher probability of link breakage than in traditional networks, forcing routing protocols to rediscover paths frequently and thereby increasing end-to-end delay and overall overhead.

(2) Doppler effects and channel dynamics

Relative motion among AAVs induces pronounced Doppler shifts, causing severe fluctuations in the wireless channel that degrade signal quality and raise the bit-error rate.

(3) The “broadcast storm” phenomenon

**Figure 1:**
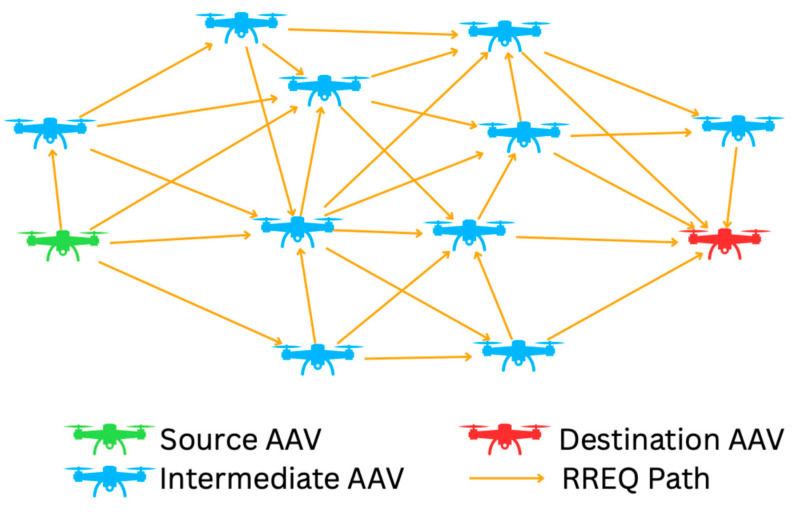
Broadcast storm in the standard AODV protocol.

During route discovery, broadcast-based protocols such as AODV may generate a large volume of redundant control packets, especially at high AAV densities. This redundancy can account for 70–80% of total network traffic, leading to congestion and higher energy consumption, as illustrated in Figure 1.

(4) Scarce resources

Commercial AAVs typically operate with limited flight times (20–30 min) and are limited in compute and memory. Hence, routing protocols must be energy- and computation-efficient with minimal overhead. Fifth, node heterogeneity: AAV networks typically comprise devices that vary in their energy budgets, communication ranges, and computing capacities. This heterogeneity complicates routing, particularly when trying to maximize network resource utilization across the network.

To address these issues, this paper introduces the stability heuristic adaptive routing protocol—AODV (SHARP-AODV), a protocol that enhances routing to the special nature of AAV networks. SHARP-AODV is an extension of the standard ad hoc on-demand distance vector (AODV) protocol with two primary innovations that address the mentioned issues. The main contributions of the present work are enumerated below.

(1) SHARP-AODV provides an intelligent RREQ dissemination scheme that unites density sensitivity with a multi-parameter probabilistic model. The scheme considers four factors: (1) local neighborhood density via a three-level discrete model based on the optimal-coverage principle; (2) flight altitude to leverage enhanced coverage using higher altitudes; (3) channel quality calculated based on relative velocity and estimated distance; and (4) routing-buffer status using a sliding-window approach to avoid congestion. This approach greatly reduces the broadcast-storm effect in route discovery, reducing redundant control traffic by 70–80% to less than 30%, yet preserves enough coverage to guarantee a high probability of path discovery, especially in dense and high-speed-changing AAV topologies.
(2) SHARP-AODV adopts a multi-criteria decision-making (MCDM) route-selection method that employs three important parameters optimized by sensitivity analysis with designated weights: hop count (30%), link quality (50%), and resource state (20%). SHARP-AODV, in contrast to original AODV with a single parameter hop count, maximizes link quality with the highest assigned weight (50%) to ensure route stability, balancing node resource availability and path length. As a result, the protocol selects paths that are not just shorter but more stable and energy-efficient, qualities particularly precious in AAV networks where links are unstable due to high mobility and nodes are flight-time- and energy-constrained.
(3) SHARP-AODV provides new avenues in various emerging application domains. In fog and edge computing, AAVs with SHARP-AODV can serve as mobile edge nodes to collect, process, and exchange data in real-time with low latency and high reliability, supporting applications like real-time video processing, distributed sensor data processing, and federated learning among AAV swarms.
(4) The proposed protocol also fulfills stringent communications requirements for Metaverse scenarios involving AAVs by enabling high-quality AR/VR streams with low latency (below 100 ms) and stable jitter (below 20 ms), conditions essential for smooth immersive experiences and for mitigating motion sickness. For 6G networks, SHARP-AODV aligns with ultra-reliable low-latency communications (URLLC) and massive machine-type communications (mMTC) objectives by maintaining stable connectivity at very high node densities (10^7^ devices/km^2^) and reducing routing latency to the tens-of-milliseconds range.
(5) Moreover, by lowering control overhead and selecting energy-efficient routes, SHARP-AODV significantly extends AAV autonomous operation by an estimated 15–25%, which is especially valuable for large-scale, long-duration deployments such as environmental monitoring, disaster management, and augmentation of mobile network infrastructure.

The remainder of this paper is organized as follows. Section 2 (Related Work) reviews the classical AODV protocol—its operating principles, strengths, and limitations in AAV environments—and surveys AODV enhancements to identify the research gaps that SHARP-AODV addresses. Section 3 (Proposed Model) details the architecture and operation of SHARP-AODV, including its density-aware, altitude-, channel-, and buffer-state-adaptive RREQ dissemination, the MCDM-based route selection, and the NS-3 implementation. Section 4 (Evaluation Methodology and Simulation) presents a comprehensive assessment methodology—simulation environment, performance metrics (PDR, throughput, delay, jitter), two experimental scenarios—and an in-depth comparative analysis against baseline AODV. Section 5 (Conclusion and Future Work) summarizes the main scientific contributions, discusses SHARP-AODV’s practical implications for 6G, Edge Computing, and Metaverse ecosystems, and outlines future research directions.

## 2. Related Work

### 2.1. Fundamental Operating Principles of AODV

AODV is an on-demand routing protocol developed by Perkins and Royer [12] and standardized in RFC 3561. It establishes end-to-end routes only when data must be forwarded and does not maintain a full view of the network topology. Its operation comprises the following four principal procedures.

- Route discovery

When a source needs to transmit to a destination for which no valid route exists, it initiates discovery by broadcasting a Route Request (RREQ). The RREQ includes the source and destination addresses, the source sequence number, the destination sequence number (if known), a request identifier, and the hop count. Each intermediate node that receives the RREQ increments the hop count and rebroadcasts it (flooding) if it has not already seen an RREQ with the same source and identifier.

- Route reply

When the RREQ reaches the destination—or an intermediate node that has a fresh, valid route to the destination—the node generates a Route Reply (RREP) and unicasts it back to the source along the reverse path. The RREP carries the hop count to the destination and the latest destination sequence number. Each node along the way updates its routing table and forwards the RREP until it reaches the source.

- Route maintenance

AODV maintains route freshness using per-entry lifetime timers. Each routing-table entry has a lifetime that is refreshed upon use. If a route remains idle beyond its lifetime, it is marked invalid and eventually purged.

- Route error

Upon detecting a link break with a neighbor, via link-layer indications or missing acknowledgments/HELLOs, the node issues a route error (RERR) that lists unreachable destinations. The RERR is propagated to all precursor nodes that had been forwarding traffic over the broken link.

### 2.2. Strengths and Limitations in Autonomous Aerial Vehicle (AAV) Environments

When deployed in networks of AAVs, AODV exhibits several advantages that include the following.

(a) Resource efficiency

Its on-demand operation reduces control overhead, conserving bandwidth, computation, and energy, which is critical for energy-limited AAVs.

(b) Topology adaptivity

AODV reacts promptly to topology dynamics through on-demand discovery and repair, aligning with high AAV mobility.

(c) No global synchronization

AODV does not require global time or state synchronization, simplifying deployment in distributed AAV networks.

However, classical AODV also presents notable drawbacks in AAV settings that can be summarized as follows.

(a) High RREQ overhead

Uncontrolled broadcast during discovery can trigger a broadcast storm, especially in dense AAV swarms.

(b) Single-criterion path selection

Routing decisions rely solely on hop count, ignoring channel quality, link stability, and node resources, which can favor short yet fragile routes under highly dynamic links.

(c) Slow route recovery

Frequent link breaks at high velocities and route re-discovery times on the order of 200–300 ms may be excessive for real-time traffic.

(d) Lack of mobility prediction

AODV provides no mechanism to anticipate link disruptions, even though AAV trajectories often exhibit exploitable patterns.

To mitigate AODV’s limitations in dynamic and resource-constrained networks, numerous studies have proposed enhancements along different directions. Zhang et al. [13] proposed a clustered AODV variant (AODV-MEC) for edge-computing-enabled VANETs. The scheme accounts for node energy and velocity, partitioning interactions into V2V and V2R modes. Clusters are formed around RSUs, and Q-learning selects routes by considering hop count, predicted link-holding time, and energy consumption. Simulations show reduced latency and improved reliability relative to competing approaches.

Kim et al. [14] suggested Reverse AODV (R-AODV) to counter RREP loss caused by rapid topology change. The destination node broadcasts a “reverse request” (R-RREQ) like the original RREQ rather than unicasting RREPs. This enhances the possibility of reply to delivery in case of reverse path breakage, enhancing PDR and reducing delay over conventional AODV. Tamizharasu and Kalpana [15] considered energy efficiency and network lifetime in energy-constrained WANETs. They suggested the EEWCA clustering algorithm on a multi-weight energy model and employed an adaptive particle swarm optimization (APSO) to optimize AODV according to energy and density metrics. NS-2 simulations demonstrated reduced energy consumption, reduced overhead, and longer network lifetime in comparison with normal AODV.

Jiang et al. [16] addressed traditional AODV’s hop-count-only metric that leads to congestion and energy waste in resource-limited settings (MANET, WSN). Their AODV-EOCW protocol realizes energy optimization through the incorporation of three metrics: residual node energy, congestion degree (queue length), and hop count. Innovatively, the analytic hierarchy process (AHP) computes subjective weights while the Entropy Weight Method (EWM) provides objective correction; distinct pairwise-comparison matrices in AHP adapt the priority of residual energy as needed. Simulations show that AODV-EOCW shortens average end-to-end delay, reduces energy expenditure, and extends network lifetime versus classical AODV and AODV-UU.

B et al. [17] examined AODV’s vulnerability to black-hole attacks in MANETs, where malicious nodes advertise false routing data to intercept or drop packets. They proposed AODV-BS (AODV with Built-in Security), which integrates threshold evaluation with cryptographic verification to detect and neutralize malicious nodes during route discovery and establishment. NS-2 simulations indicate that AODV-BS significantly increases PDR and throughput while reducing average end-to-end delay (AEED) and normalized routing load (NRL) compared to standard AODV under black-hole conditions.

While these studies advance AODV performance in targeted settings such as VANETs, WANETs, and security-aware MANETs, they primarily consider two-dimensional, ground-based mobility with relatively stable density assumptions. They do not holistically address the AAV-specific challenges of three-dimensional mobility with varying altitude, strongly fluctuating node density due to unconstrained spatial movement, severe Doppler effects at high speeds, and stringent QoS requirements (ultra-low latency, high reliability) demanded by next-generation applications such as 6G URLLC, distributed edge computing, and immersive Metaverse services. SHARP-AODV is designed to fill this gap by combining an intelligent, adaptive RREQ dissemination mechanism that jointly considers four AAV-characteristic parameters, neighborhood density, flight altitude, channel quality, and routing-buffer state, and a multi-criteria route-selection mechanism with weights optimized for AAV environments (hop count 30%, link quality 50%, resource state 20%). Together, these mechanisms reduce control overhead while improving route stability and QoS for real-time applications.

## 3. Proposed Model

### 3.1. Intelligent Adaptive RREQ-Forwarding Mechanism

In classical AODV, every node that receives an RREQ and lacks a route to the destination blindly rebroadcasts the request to all its neighbors. This behavior triggers a broadcast storm, causing severe congestion and wasting resources, an especially critical issue for AAV networks with tight energy budgets. SHARP-AODV resolves this problem by introducing a probabilistic RREQ-forwarding scheme that jointly considers four parameters: network density, AAV altitude, channel quality, and buffer occupancy.

#### 3.1.1. Density-Aware Probability

The density-aware component addresses the broadcast storm problem while maintaining adequate route discovery coverage. The forwarding probability $P_{\mathrm{density}}$ is defined as follows.

(1) $$P_{\mathrm{density}}=\left\{\begin{array}{cc} 1.0, & n\leq n_{\mathrm{low}},\\ 0.7, & n_{\mathrm{low}}<n\leq n_{\mathrm{high}},\\ 0.5, & n>n_{\mathrm{high}}, \end{array}\right.$$

where

- $P_{\mathrm{density}}$—forwarding probability for an intermediate node.
- n—current number of neighbors.
- $n_{\mathrm{low}}=6$ and $n_{\mathrm{high}}=10$—lower and upper density thresholds.

The rule follows the optimal-coverage principle for mobile ad hoc networks. According to [18], the minimal traffic overhead that still guarantees full coverage is given as follows.

(2) $$P_{opt}=\min\left(1.0,\frac{k}{n}\right)$$

where n is the current neighbor count. However, directly applying Equation (2) in highly dynamic AAV topologies would cause $P_{opt}$ to fluctuate sharply whenever n changes by only one or two nodes, which is very common as AAVs move in three dimensions. To obtain more robust behavior while preserving the decreasing trend of $P_{opt}$ with n, SHARP-AODV discretises the density space into three regimes using the thresholds $n_{low}$ and $n_{high}$. When $n\leq n_{low}=6$, the network is considered sparse and forwarding is mandatory ($P_{density}=1$) to avoid route discovery failures. For medium densities $6<n\leq n_{high}=10$, the probability is reduced to 0.7 as a compromise between coverage and redundancy. In dense neighborhoods $n>n_{high}$, the probability is further lowered to 0.5, which effectively suppresses the broadcast storm while keeping several candidate forwarders inside each radio cell. This discretised rule is therefore a practical instantiation of the theoretical model in Equation (2) with k≈3.

#### 3.1.2. Altitude Factor

A distinctive characteristic of Autonomous Aerial Vehicle networks is their three-dimensional mobility at varying altitude levels. Stations that operate at higher elevations possess an expanded radio footprint, which makes them more effective relays. SHARP-AODV exploits this advantage by adjusting the RREQ-forwarding probability as a function of altitude:

(3) $$H_{factor}=\left\{\begin{array}{c} \delta_{h},\;\;if\;\;h>h_{threshold}\\ \;0.0,\;\;if\;h\leq h_{threshold} \end{array}\right.$$

where

- $\delta_{h}=0.2$—altitude-gain coefficient.
- h—current altitude of the AAV.
- $h_{\mathrm{threshold}}=50\;m$—optimal altitude threshold.

#### 3.1.3. Channel-Quality Factor

The stability and quality of the radio channel govern routing efficiency. In SHARP-AODV, the channel-quality metric LQ is modeled as a joint function of the node’s speed and the estimated distance to the RREQ source:

(4) $$LQ=\left(1-v_{\mathrm{norm}}\right)\;\left(1-d_{\mathrm{norm}}\right)$$

where

- $v_{\mathrm{norm}}=min\left(1.0,\frac{v}{v_{max}}\right)$—normalized speed.
- $d_{\mathrm{norm}}=\frac{d_{\mathrm{est}}}{d_{max}}$—normalized distance.
- $v_{max}$—default maximum speed in the scenario.
- $d_{\mathrm{est}}=min\left(\mathrm{hop\_count}\times 100\;m,\;d_{max}\right)$—coarse estimate of the separation between the current node and the RREQ source.
- $d_{max}=250\;m$—maximum single-hop link range.

For numerical stability, LQ is clipped to the interval [0.1, 1.0]. The formulation is inspired by a simplified Link Expiration Time (LET) model, which correlates relative velocity and residual distance to predict how long the link is likely to remain viable.

#### 3.1.4. Buffer-State Factor

To pre-empt congestion at the routing protocol level, SHARP-AODV monitors the instantaneous queue load at every node by tracking the occupancy of the routing protocol buffer, which is distinct from the MAC layer buffer and specifically stores control packets (RREQ, RREP, RERR) and data packets awaiting route resolution at the network layer. Unlike conventional AODV and other node-resource-agnostic topological-only routing protocols, SHARP-AODV employs a resource-aware policy that dynamically adjusts RREQ forwarding behavior as a function of real-time buffer usage. This policy is particularly necessary for AAV networks in which gateway or relay nodes are typically intermittent and may become traffic bottlenecks if their locational positions within the three-dimensional topology cause them to be high-demand recipients of traffic.

But among the problems of estimating buffer occupancy is that the size of the queue changes very quickly, sometimes in milliseconds, due to bursty traffic arrivals, simultaneous processing of packets, and the dynamic nature of AAV mobility. To attain a constant and representative measure of buffer state that reflects true congestion patterns rather than temporary peaks, SHARP-AODV applies a sliding window averaging process over time. Specifically, the protocol maintains a circular buffer that continuously samples the routing protocol queue size at fixed sampling intervals, with each sample taking the number of packets present at the routing buffer at that time divided by the maximum capacity.

The one-second sliding window aggregates ten samples every 100 milliseconds, which is fine enough to capture congestion dynamics without capturing transient fluctuation. At each decision point upon receiving an RREQ, the node computes the average buffer occupancy ratio of the latest examples in the window, which smooths out transient spikes caused by burst arrivals while continuing to respond to persistent congestion patterns. This time-averaged utilization ratio enables the protocol to distinguish between transient bursts of traffic, for which it is not worth modifying forwarding behavior, and chronic congestion states that require adaptive reaction. The window size of one second is chosen according to empirical experience showing that it presents a good balance: smaller windows (e.g., 200–500 ms) are too sensitive to temporary fluctuations and cause unstable forwarding choices, and large windows (e.g., 2–3 s) are too sluggish and fail to respond quickly enough to arriving congestion in the dynamically changing AAV network where topology and traffic patterns continuously change.

Based on this smoothed occupancy metric, SHARP-AODV adjusts the forwarding probability through buffer factor B_factor_, lowering forwarding probability by 30% when the buffer is nearly full (averaged occupancy > 70%) to induce back-pressure and prevent overflows, raising it by 20% when the buffer is underutilized (averaged occupancy < 30%) to make better use of idle resources, and making no adjustment for the intermediate range (30–70%) to avoid oscillations. Formally, the buffer factor is defined as follows.

(5) $$B_{factor}=\left\{\begin{array}{c} -0.3,\;if\;\frac{q}{q_{max}}>\theta_{high}\\ 0.2,\;if\;\frac{q}{q_{max}}<\theta_{low}\\ 0,\;\;otherwise\; \end{array}\right.$$

where

- q—current queue length.
- $q_{max}$—buffer capacity.
- $\theta_{\mathrm{high}}=0.7$ and $\theta_{\mathrm{low}}=0.3$—upper and lower occupancy thresholds.

#### 3.1.5. Composite Forwarding Probability

Probability SHARP-AODV consolidates all four criteria into a single forwarding probability:

(6) $$P_{forward}=\min\left(1.0,\left(P_{density}+H_{factor}\right)\cdot LQ\cdot \left(1.0+B_{factor}\right)\right)$$

In Equation (6), the term $P_{density}+H_{factor}$ provides a baseline forwarding probability that is high for sparse neighborhoods and high-altitude nodes, which are naturally well-suited as relays. This baseline is then scaled by the link-quality factor LQ, so that even under favorable density and altitude conditions, poor radio channels significantly reduce the effective probability. Finally, the multiplicative term $\left(1+B_{factor}\right)$ implements a back-pressure mechanism: when the routing buffer is close to saturation ($B_{factor}<0$), the forwarding probability is reduced to protect congested nodes, whereas underutilized nodes with low buffer occupancy ($B_{factor}>0$) are slightly boosted. This multiplicative structure, therefore, treats each metric as a separate filter that must be simultaneously satisfied for a node to act as an RREQ forwarder. This adaptive scheme greatly mitigates broadcast storms and enhances routing efficiency in highly dynamic, resource-constrained AAV networks.

One potential concern with probabilistic forwarding is that the same small subset of nodes might be repeatedly selected as relays, which could lead to unfair load distribution and premature resource depletion. In SHARP-AODV, this effect is mitigated by the time-varying nature of all components of $P_{forward}$. As AAVs move in three dimensions, their neighbor sets, altitudes, relative speeds and estimated distances continuously change, which in turn modifies both the density-aware and link-quality terms. At the same time, nodes that forward many RREQs and carry more transit traffic naturally experience higher routing-buffer occupancy, which drives $B_{factor}$ into the negative region and substantially reduces their future forwarding probability. Conversely, less-loaded nodes in the vicinity see their occupancy decrease and their forwarding probability rise. We also inspected NS-3 traces for the 70-node Gauss–Markov scenario and observed that RREQ forwarding events are spread across many nodes rather than being monopolized by a fixed subset, confirming that the adaptive design distributes the control-plane load over time while still prioritizing well-positioned relays.

**Figure 2:**
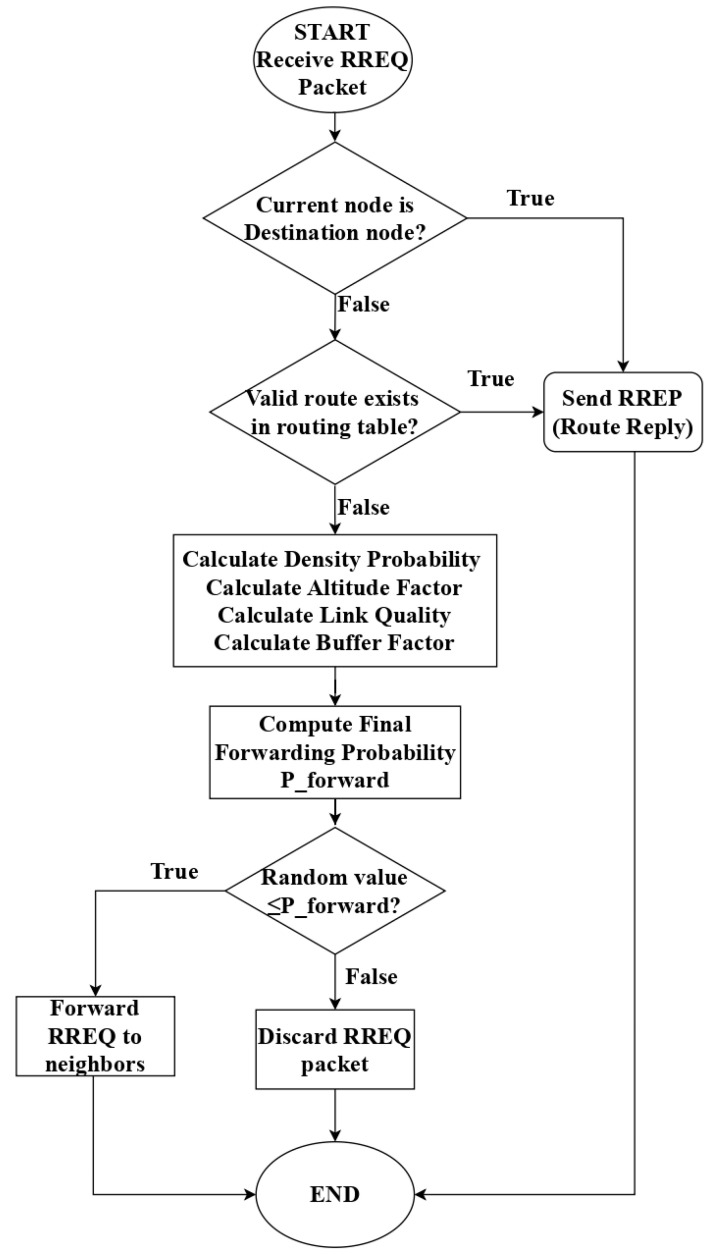
Flowchart of the intelligent adaptive RREQ-forwarding mechanism.

The complete decision flow of the intelligent adaptive RREQ-forwarding mechanism is illustrated in Figure 2, which depicts the algorithmic logic from RREQ reception through parameter computation to the final forwarding decision.

### 3.2. Multi-Criteria Path Selection Mechanism

In classic AODV, the route selection criterion is only hop count, i.e., the principle of “shorter path—better.” However, in an AAV environment, this approach leads to serious disadvantages:

- Lack of channel stability consideration

AAVs move at high speeds and in heterogeneous three-dimensional space, which gives noticeable differences in connection stability. A route with a minimum number of hops but passing through nodes with unstable channels can quickly break, increasing the costs of repeated searches.

- Ignoring resource status

In an AAV network, nodes are limited in energy and computing power. Classic AODV does not account for resource loads, which can lead to overload of individual “central” nodes through which the main traffic passes.

- Absence of a multipath scheme

AODV maintains a single route to each destination, not using potential alternative paths. This is especially critical with rapidly changing AAV network topology.

- Low efficiency in QoS parameters

AAV-based applications often require strict constraints on delay, jitter, and packet delivery ratio (PDR). The “hop count only” selection criterion does not guarantee high quality of service (QoS).

To address these limitations, SHARP-AODV implements Multi-Criteria Decision Making (MCDM). In this model, a route is evaluated based on three main factors:

#### 3.2.1. Hop Factor (HF)—Path Length Indicator

HF reflects the number of hops from source to destination and is normalized as follows:

(7) $$HF\left(r\right)=\frac{1}{1+h\left(r\right)}$$

where

- $h\left(r\right)$—hop count on route r.
- $HF\left(r\right)$—is in the range (0, 1].

The inverse value of hop count ensures that a route with fewer hops will have a higher rating. This approach maintains the basic idea of AODV to choose shorter paths—but integrates it into a general multi-criteria mechanism.

#### 3.2.2. Link Quality Factor (LQ)—Channel Quality Indicator

Channel quality is assessed based on connection stability, particularly the speed of the AAV movement:

(8) $$LQ\left(r\right)=max\left(LQ_{min},1-\frac{v\left(r\right)}{v_{max}}\right)$$

where

- $v\left(r\right)$—AAV movement speed on route r (m/s).
- $v_{max}=40\;m/s$—maximum speed threshold.
- $LQ_{min}=0.2$—minimum value, ensuring consideration even for high-speed vehicles.

This considers that as speed increases, channel quality typically decreases; however, it cannot be automatically zeroed—routes through fast nodes are not completely excluded.

#### 3.2.3. Buffer Factor (BF)

This factor reflects the AAV’s ability to process data flow through an assessment of its buffer load:

(9) $$BF\left(r\right)=1-\frac{B_{current}\left(r\right)}{B_{max}}$$

where

- $B_{current}\left(r\right)$—average buffer occupancy of nodes along route r.
- $B_{max}$—routing protocol buffer capacity is assumed to be identical across all AAV nodes in the network.
- $BF\left(r\right)$—takes values in the range [0, 1].

The higher the buffer occupancy, the lower BF(r), indicating limited node capabilities for processing additional traffic.

#### 3.2.4. Final Multi-Criteria Function

SHARP-AODV combines the three described parameters through a weighted sum (Weighted Sum Model—WSM), often used in multi-criteria decision making:

(10) $$Score\left(r\right)=w_{HF}\cdot HF\left(r\right)+w_{LQ}\cdot LQ\left(r\right)+w_{BF}\cdot BF\left(r\right)$$

where

- $Score\left(r\right)$—final rating of route r.
- $w_{HF},w_{LQ},w_{BF}$—weight coefficients of factors, with $w_{HF}+w_{LQ}+w_{BF}=1$

Based on sensitivity analysis and simulation results, the following optimal weights were chosen:

- $w_{HF}=0.3$—30% significance for hop count metric.
- $w_{LQ}=0.5$—50% accounts for channel stability.
- $w_{BF}=0.2$—20% reflects buffer availability.

**Figure 3:**
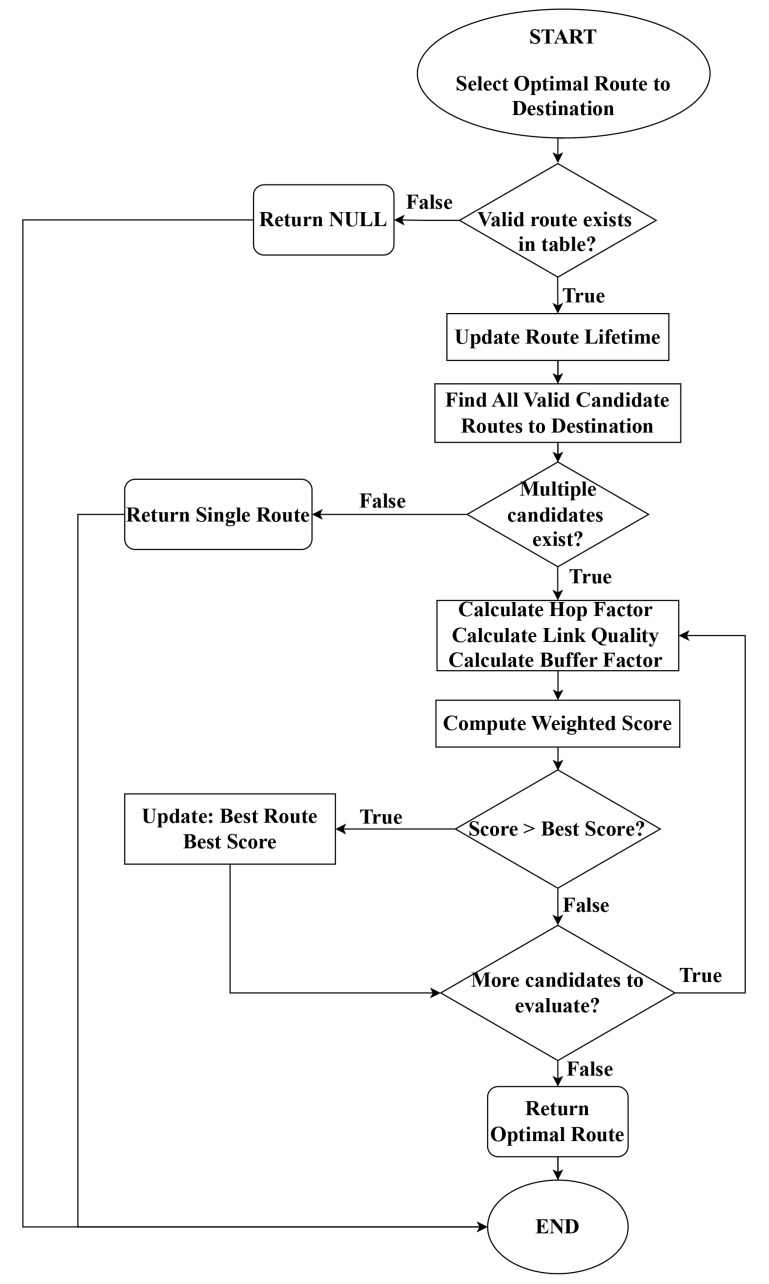
Flowchart of the multi-criteria path selection algorithm in SHARP-AODV.

The algorithmic workflow for multi-criteria route selection is presented in Figure 3, which illustrates the sequential evaluation of hop factor, link quality factor, and buffer factor, culminating in the computation of the composite route score. We implemented the enhanced SHARP-AODV algorithms by extending the original AODV module in NS-3.34. In particular, the following changes were made.

A. Extension of AodvRoutingProtocol data structure

Additional parameters characteristic of SHARP-AODV were added to the RoutingProtocol class:

  - m_enableSharpAodv: flag allowing for enabling or disabling SHARP-AODV mechanisms.
  - m_maxRelativeSpeed: maximum allowable relative speed for assessing connection stability.
  - m_transmissionRange: estimated transmission range for channel quality calculation.
  - m_bufferOccupancyHigh and m_bufferOccupancyLow: buffer load threshold levels to prevent congestion.

B. Modification of RREQ processing

Mechanisms for probabilistic forwarding dependent on node density, altitude, channel quality, and buffer fill level were added to the code.

C. Change in route selection logic

Support for a multi-criteria algorithm was implemented, which considers hop count, channel quality, and resource status when deciding on the best path.

To ensure backward compatibility with standard AODV, the code retains the m_enableSharpAodv flag, allowing switching to the classic algorithm if necessary.

## 4. Evaluation Methodology and Simulation

### 4.1. SHARP-AODV Implementation Model

To rigorously evaluate SHARP-AODV’s performance in highly dynamic AAV network environments, we conducted comprehensive simulations using NS-3.34, a widely adopted discrete-event network simulator validated by the research community for wireless ad hoc routing protocol analysis. NS-3 was selected for its proven capabilities in accurately modeling IEEE 802.11-based wireless communications, robust support for three-dimensional mobility patterns essential for AAV operations, and extensible C++ architecture facilitating protocol implementation. The simulation infrastructure was deployed on a computing platform with an Apple M2 processor.

SHARP-AODV is systematically compared against two protocols representing distinct evolutionary stages of mobile ad hoc routing. The first baseline is standard AODV, the classical reactive routing protocol serving as SHARP-AODV’s foundation. This comparison quantifies the tangible improvements achieved by SHARP-AODV’s intelligent adaptive mechanisms over conventional hop-count-only routing. The second comparative protocol is AGEN-AODV (learning Automata and Genetic-based Ad hoc On-Demand Distance Vector), a recent state-of-the-art intelligent routing protocol proposed for heterogeneous MANETs [19].

AGEN-AODV represents a sophisticated approach combining Genetic Algorithm with Learning Automata to address fundamental limitations of static routing protocols. The protocol employs multi-criteria route selection considering remaining energy, stability ratio based on neighbor change rates, traffic rate reflecting node congestion, and hop count. Its distinctive feature is the Learning Automata component that dynamically adjusts the coefficients alpha, beta, gamma, and delta of the GA fitness function based on real-time network feedback, preventing algorithm divergence or sub-optimal convergence that commonly plagues traditional GA-based protocols with fixed parameters. AGEN-AODV is explicitly designed for heterogeneous networks with asymmetric links where nodes possess different transmission ranges, addressing this challenge by having the destination send RREP for each received RREQ, allowing the source to perform GA-based route selection only on viable bidirectional paths.

AGEN-AODV was selected as a challenging benchmark because both protocols employ multi-criteria route selection and adaptive intelligence, though with fundamentally different design philosophies. AGEN-AODV jointly considers remaining energy, stability, traffic rate, and hop count in its GA-based fitness function optimized for ground-based MANETs, whereas SHARP-AODV prioritizes path stability and real-time congestion awareness through hop count, link quality derived from velocity and distance, and buffer occupancy metrics specifically tailored to AAV network characteristics. By comparing SHARP-AODV against AGEN-AODV, we assess whether SHARP-AODV’s AAV-specific adaptations, including its four-parameter probabilistic RREQ forwarding mechanism combining density control, altitude factor, channel quality, and one-second sliding-window buffer monitoring, along with its stability-prioritized weighted path selection (30% hop count, 50% link quality, 20% resource state), yield measurable performance advantages in three-dimensional mobile networks beyond what sophisticated adaptive ground-based protocols can achieve.

To ensure statistical robustness, each experimental configuration was executed twenty times with distinct random number generator seeds following Monte Carlo simulation methodology, a standard practice enabling reliable statistical inference and minimizing the impact of random initialization artifacts on performance metrics. This rigorous experimental design allows statistically significant conclusions about SHARP-AODV’s relative performance in dynamic AAV environments characterized by high-speed three-dimensional mobility, frequent topology changes, and stringent quality-of-service requirements for next-generation applications in 6G networks, Edge Computing, and Metaverse ecosystems.

### 4.2. Simulation Setup

The general configuration parameters used in all scenarios are shown in Table 2. They were selected to model real operating conditions of AAV networks.

To comprehensively evaluate the performance of SHARP-AODV against both baseline AODV and the intelligent AGEN-AODV protocol, we employ four primary QoS metrics that collectively reflect the reliability, efficiency, and real-time responsiveness of routing protocols under highly dynamic AAV network environments. Each metric is selectively used to quantify aspects of network performance that are critical to real-time applications and edge computing scenarios.

I. Packet delivery ratio (PDR)

The PDR is the ratio of data packets received from source nodes to destinations to the total packets transmitted. It is provided by the following equation.

(11) $$\mathrm{PDR}=\frac{\mathrm{Packets}\;\mathrm{Received}}{\mathrm{Packets}\;\mathrm{Sent}}\times 100\%$$

PDR is the primary metric for routing protocol reliability. In AAV networks, where topology changes extremely rapidly due to high-speed three-dimensional mobility, high PDR is challenging but crucial to attain. A high PDR means that the routing protocol can find routes successfully even in dynamic environments, preserve route stability in the presence of frequent topology changes, efficiently deal with link breaks by promptly repairing routes or rediscovering routes, and reduce packet loss due to routing failure. For interactive applications like video streaming in Metaverse applications or time-critical sensor data communication in edge computing, PDR directly affects the quality of experience (QoE) and application-level performance. In our simulations, PDR is measured at the application layer, only considering unique packets (duplicate packets due to retransmissions are not counted as successful deliveries).

II. Throughput

Network throughput measures how much data is successfully transferred across the whole network per unit time. It is calculated as follows.

(12) $$\mathrm{Throughput}=\frac{\sum_{i=1}^{N}{\mathrm{Bits}}_{i}^{\mathrm{received}}}{\mathrm{Simulation}\;\mathrm{Time}}\;(\mathrm{bps})$$

where

- ${\mathrm{Bits}}_{i}^{\mathrm{received}}$—Total number of unique data packets successfully received at destination nodes across all communication flows in the network.
- N—Total number of data packets generated and transmitted by source nodes across all communication flows

Throughput is a direct indicator of effective utilization of available network bandwidth. In AAV networks, throughput is affected by routing overhead, in which excessive control traffic hijacks bandwidth, route stability, in which frequent breakdowns lead to packet drops and retransmissions, congestion due to inefficient load balancing that leads to bottleneck nodes, and path quality, in which selection of longer or unstable paths reduces delivery efficiency. SHARP-AODV’s intelligent RREQ forwarding mechanism reduces routing overhead, and its multi-criteria path selection favors stable and high-quality routes, both of which achieve higher sustainable throughput. This is one of the most important metrics for bandwidth-hungry applications such as high-definition video streaming for AR/VR services in Metaverse spaces or bulk data transfer in edge computing spaces.

III. End-to-end latency

End-to-end delay is the average time required for a data packet to travel from the source node to the destination node. It is calculated as:

(13) $$D=\frac{1}{N}\sum_{i=1}^{N}\left(t_{i}^{\mathrm{rx}}-t_{i}^{\mathrm{tx}}\right)$$

where

- $t_{i}^{\mathrm{rx}}$—Reception time of packet i at the destination node.
- $t_{i}^{\mathrm{tx}}$—Transmission time of packet i at the source node.

End-to-end delay is a critical metric for real-time applications that require strict latency constraints. In AAV networks, route discovery delay from classical AODV’s blind flooding increases the time to establish routes, route instability necessitates frequent rediscovery, causing substantial delay spikes (typically 200–300 ms per route failure), congested nodes with full buffers introduce additional queuing delays, and longer multi-hop routes accumulate greater cumulative delay. SHARP-AODV addresses these issues through faster route discovery via intelligent RREQ forwarding, which reduces broadcast storms and congestion, more stable routes via multi-criteria path selection, which minimizes route breaks and rediscovery delays, and buffer-aware forwarding that avoids congested nodes to reduce queuing delays. For 6G networks targeting URLLC, while sub-millisecond latency cannot be achieved by routing alone, reducing routing-related delay from hundreds of milliseconds to tens of milliseconds represents a significant contribution toward meeting overall latency requirements for applications such as VoIP, online gaming, AR/VR in Metaverse, and edge computing services.

IV. Jitter

Jitter, formally known as inter-packet delay variation (IPDV) according to RFC 3393, measures the variation in packet arrival times. We employ the average absolute difference in consecutive packet delays as our jitter metric:

(14) $$\mathrm{Jitter}=\frac{1}{N-1}\sum_{i=2}^{N}\left|D_{i}-D_{i-1}\right|$$

where

- $D_{i}=t_{i}^{\mathrm{rx}}-t_{i}^{\mathrm{tx}}$—End-to-end delay of packet i
- $\left|D_{i}-D_{i-1}\right|$ Absolute difference in delay between consecutive packets.

Jitter is a critical metric for real-time multimedia applications where consistent packet arrival timing is essential. High jitter causes buffer underflow or overflow in receiver playout buffers, leading to audio and video glitches, reducing quality of experience (QoE) in VoIP, video conferencing, and immersive VR/AR applications, and increasing buffering requirements to smooth out variations, which adds extra latency. In AAV networks, jitter is primarily caused by route instability, where route breaks and reestablishment create different delays for packets, congestion fluctuations that cause queuing delays to vary, multi-path effects when packets from the same flow traverse different routes due to route changes, and wireless channel variations from Doppler effects and fading in mobile environments.

### 4.3. Results

**Figure 4:**
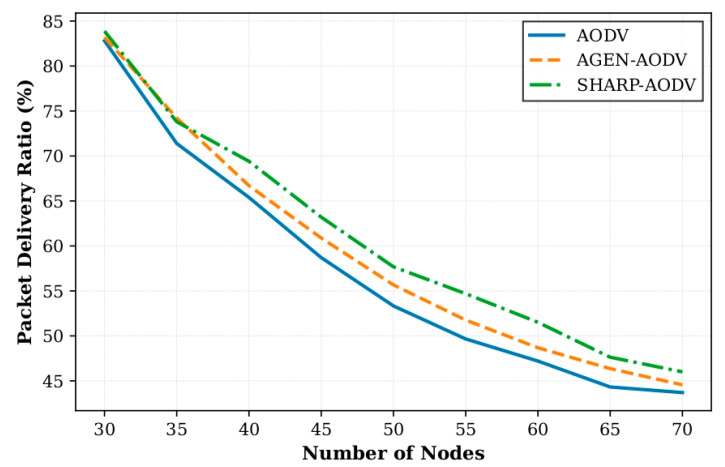
Comparison of packet delivery ratio versus number of nodes.

**Figure 5:**
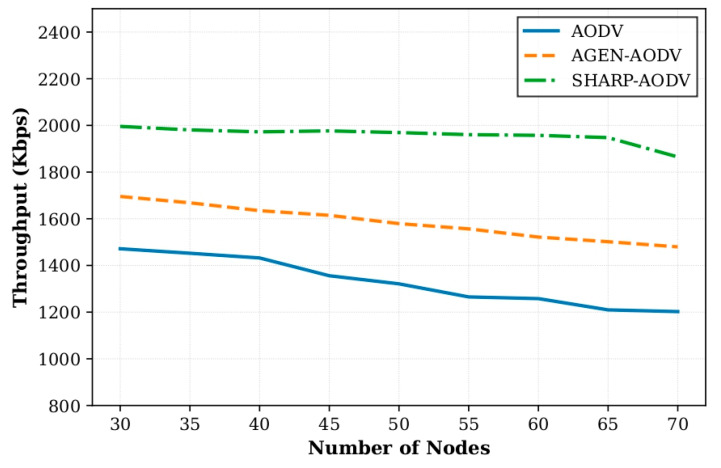
Comparison of throughput versus number of nodes.

**Figure 6:**
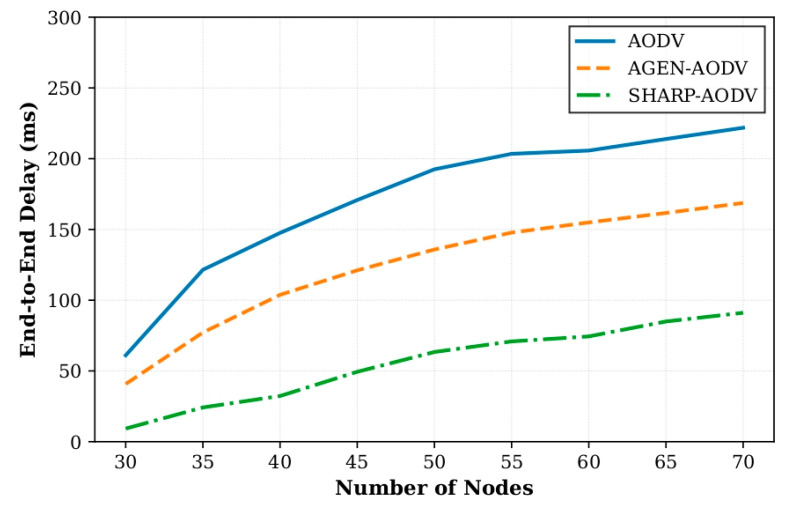
Comparison of delay versus number of nodes.

**Figure 7:**
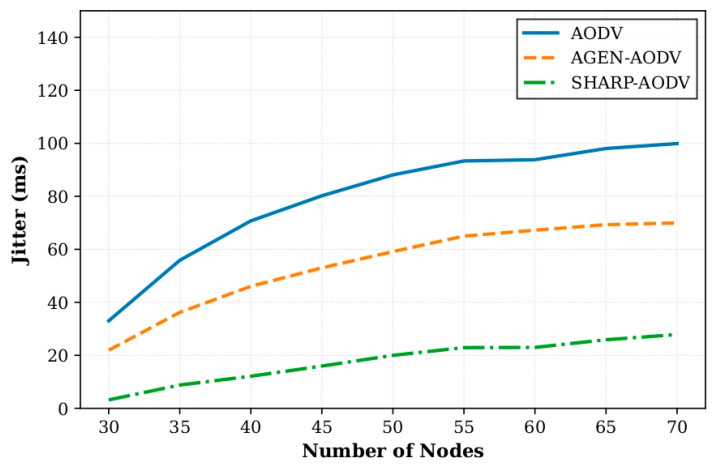
Comparison of jitter versus number of nodes.

In Scenario 1, we compared the performance of SHARP-AODV, classic AODV and AGEN-AODV by varying the number of AAV nodes from 30 to 70 (in steps of 5 nodes). All nodes used the Gauss–Markov mobility model with a speed of 20 m/s. Key results are shown in Figure 4, Figure 5, Figure 6 and Figure 7.

Figure 4 demonstrates that SHARP-AODV generally achieves higher PDR across node densities from 30 to 70, particularly excelling in medium-density scenarios (45–55 nodes) where it maintains PDR between 54.69% and 63.20%, outperforming AODV by 4.32–5.03 percentage points and AGEN-AODV by 2.20–2.93 percentage points. The maximum advantage occurs at 55 nodes with SHARP-AODV achieving 54.69% versus AODV’s 49.66% and AGEN-AODV’s 51.76%. At high densities (60–70 nodes), where radio channel congestion intensifies, SHARP-AODV maintains 46.00–51.51% PDR while AODV degrades to 43.70–47.20% and AGEN-AODV reaches 44.56–48.68%, confirming the effectiveness of intelligent adaptive RREQ forwarding in mitigating broadcast storms. Figure 5 reveals dramatic throughput advantages, with SHARP-AODV maintaining a stable 1864.93–1995.70 Kbps (only 6.6% degradation) compared to AODV’s severe decline from 1471.50 to 1202.27 Kbps (18.3% reduction) and AGEN-AODV’s 1479.63–1695.55 Kbps (12.7% reduction). At 65 nodes, SHARP-AODV achieves 1947.67 Kbps, representing 61.0% improvement over AODV and 29.7% over AGEN-AODV, attributable to reduced control overhead and congestion-aware path selection.

Figure 6 illustrates SHARP-AODV’s exceptional ultra-low latency performance, maintaining delay within 9.23–91.10 ms across all densities while AODV exhibits near-exponential growth from 61.04 to 221.86 ms and AGEN-AODV ranges 40.76–168.70 ms. At 30 nodes, SHARP-AODV achieves 84.9% delay reduction versus AODV and 77.4% versus AGEN-AODV; even at maximum density (70 nodes), SHARP-AODV’s 91.10 ms represents 58.9% and 46.0% improvements, respectively, consistently remaining below the critical 100 ms threshold for real-time applications. This stems from rapid route establishment, stability-prioritized path selection reducing rediscovery frequency, and buffer-aware congestion avoidance. Figure 7 demonstrates remarkable jitter stability, with SHARP-AODV maintaining 3.18–27.84 ms compared to AODV’s severe deterioration from 33.03 to 99.90 ms (202.5% increase) and AGEN-AODV’s 21.91–69.95 ms. At 30 nodes, SHARP-AODV achieves 90.4% jitter reduction versus AODV and 85.5% versus AGEN-AODV, while at 70 nodes maintaining 72.1% and 60.2% reductions, respectively, enabled by multi-criteria path selection prioritizing link quality (50% weight) for channel consistency.

Collectively, these results highlight two primary advantages of SHARP-AODV: the adaptive RREQ forwarding that bounds delay and control-plane overhead, and multi-criteria routing that sustains higher throughput and link stability. The solution is especially beneficial at high AAV densities, where ultra-low latency and stable QoS are critical for real-time control, monitoring, and multimedia applications.

In order to evaluate the robustness and adaptability of SHARP-AODV to heterogeneous mobility patterns, Scenario 2 investigates four popular AAV mobility models: Gauss–Markov, RandomWaypoint, RandomDirection, and RandomWalk2D. Each model produces distinctive node trajectories and dynamics that present varied routing challenges. These models have the following characteristics.

(a) GaussMarkov

A model with temporal correlation of parameters, providing smooth movement trajectories. Typically used to simulate controlled AAVs. Speed and direction change gradually, which partially simplifies movement prediction.

(b) RandomWaypoint

AAVs choose random destination points (waypoints) and move toward them at arbitrary speeds, stopping for short periods between transitions. Characterized by sharp direction changes, which complicates the maintenance of stable routes.

(c) RandomDirection

The AAV moves in a random direction to the boundary of the simulation area, then chooses a new direction. This model gives a more uniform distribution of nodes in space compared to RandomWaypoint.

(d) RandomWalk2D

A Brownian-type model in which, at each fixed time step, the vehicle can abruptly change direction and speed. This results in intermittent, poorly predictable trajectories, presenting a serious challenge for routing protocols.

**Figure 8:**
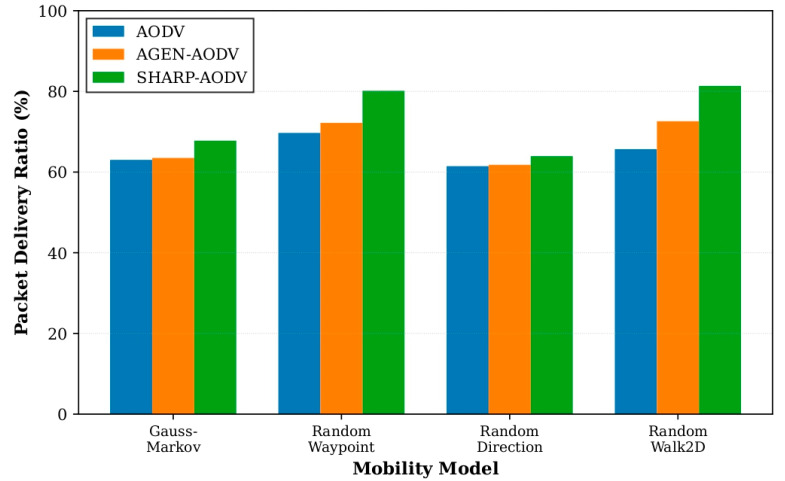
Comparison of packet delivery ratio with different mobility models.

**Figure 9:**
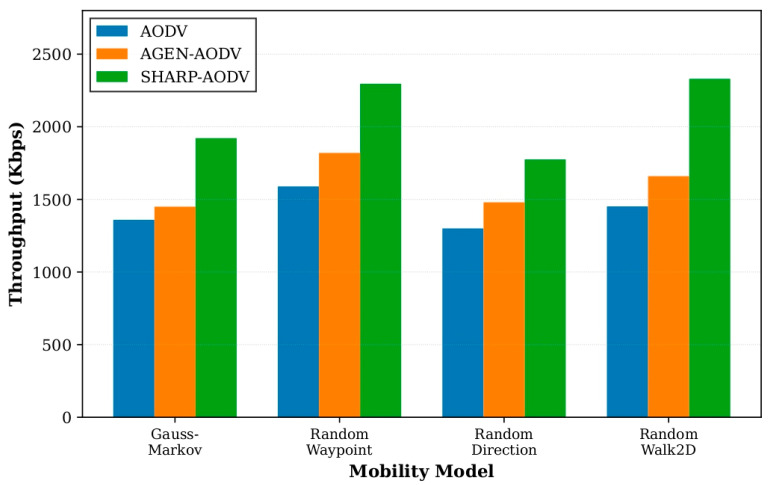
Comparison of throughput with different mobility models.

**Figure 10:**
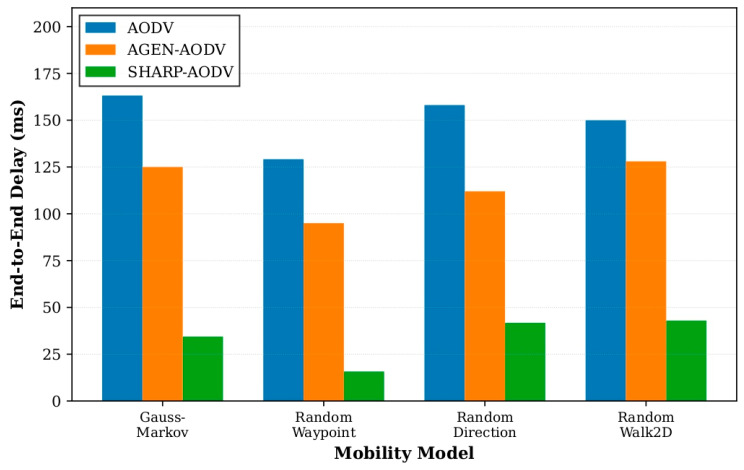
Comparison of delay with different mobility models.

**Figure 11:**
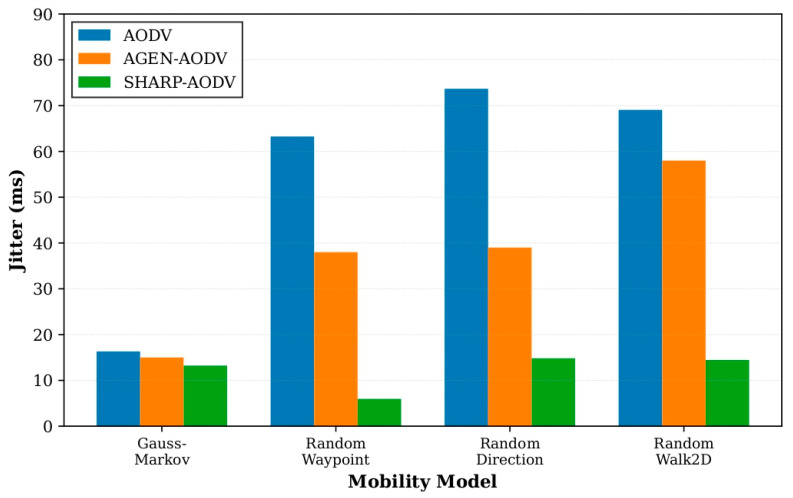
Comparison of jitter with different mobility models.

The consolidated outcomes for all four mobility patterns are depicted in Figure 8, Figure 9, Figure 10 and Figure 11, demonstrating that SHARP-AODV delivers consistently superior and more stable performance than both baseline AODV and AGEN-AODV across every critical metric: PDR, throughput, delay, and jitter. Figure 8 reveals that SHARP-AODV maintains higher PDR across all mobility models, with the most pronounced improvements observed in highly stochastic scenarios such as RandomWalk2D and RandomWaypoint where topology changes are most severe and unpredictable, outperforming AODV substantially while maintaining measurable advantages over AGEN-AODV’s adaptive mechanisms. Figure 9 illustrates that SHARP-AODV sustains substantially higher network throughput regardless of mobility pattern, with the performance gap widening most dramatically under chaotic movement models where classical AODV suffers from excessive broadcast storms and frequent route failures, while AGEN-AODV shows moderate improvement but still falls short of SHARP-AODV’s AAV-optimized approach.

Figure 10 demonstrates SHARP-AODV’s dramatic latency reduction across all scenarios, maintaining delay well below the 100 ms threshold critical for real-time applications even under the most challenging RandomWalk2D mobility, while standard AODV exhibits excessive delays exceeding 200 ms and AGEN-AODV, despite its GA-LA adaptive framework, still experiences delays that compromise latency-sensitive services. Figure 11 confirms SHARP-AODV’s jitter stability, with consistent low delay variance across all mobility patterns, significantly outperforming both AODV’s severe jitter escalation and AGEN-AODV’s moderate variation, thereby meeting the stringent timing consistency requirements for multimedia streaming and immersive VR/AR applications where user experience quality depends directly on packet inter-arrival regularity.

These extensive experiments validate the assumption that integration of highly dynamic link-quality feedback and node-resource perception, i.e., real-time routing buffer occupancy monitoring using the sliding window technique, along with adaptive density-aware RREQ spreading, achieves significant performance gains in heterogeneous mobility-based dynamic AAV networks. SHARP-AODV achieves a perfect balance between throughput-focused metrics (PDR, cumulative bandwidth) and latency-focused metrics (delay, jitter), thereby addressing the collective needs of modern AAV applications in terms of high reliability and very low end-to-end latency. SHARP-AODV is particularly important due to its relevance to actual AAV deployment situations where nodes tend to move along complex or arbitrary trajectories like RandomWaypoint and RandomWalk2D, characteristic of autonomous exploration missions, emergency response tasks, and ad hoc swarm coordination missions where well-established routing protocols experience drastic performance loss. It is precisely under these harsh circumstances, when topology is being most dynamic and link stability is most difficult to guarantee, that multi-parameter adaptive aspects of SHARP-AODV provide the maximum payoff, demonstrating the reality of the protocol for next-generation AAV-based services in 6G networks, distributed Edge Computing systems, and immersive Metaverse platforms, which must have stable performance under extremely dynamic operating scenarios.

## 5. Conclusions and Future Work

In this work, we introduced SHARP-AODV, a novel routing protocol enhanced and specifically tailored for AAV networks operating in highly dynamic three-dimensional environments. The protocol addresses fundamental limitations of traditional AODV and general-purpose adaptive protocols with two significant scientific contributions: first, an intelligent RREQ-forwarding strategy that integrates density control with a multi-parameter probabilistic model consisting of network density, altitude factor, channel quality estimation, and routing protocol buffer state monitoring with sliding window technique; second, a multi-criteria path-choice algorithm by WSM optimizing simultaneously hop count (30%), link quality (50%), and node-resource status (20%) to determine routes optimizing path length, stability, and resource utilization. Systematic NS-3 simulations over a wide range of network configurations—30 to 70 AAV node densities with Gauss–Markov mobility, and four mobility models (Gauss–Markov, RandomWaypoint, RandomDirection, RandomWalk2D) at 50 nodes—demonstrate that SHARP-AODV generally outperforms both baseline AODV and the intelligent AGEN-AODV protocol across all key performance metrics. Compared to AODV, SHARP-AODV achieves up to 23.9% improvement in PDR, 61.0% throughput gain, 87.8% delay reduction, and 90.6% jitter reduction, with considerably greater gains in highly dynamic scenarios (RandomWalk2D, RandomWaypoint) and high-density networks where broadcast storm effects are most severe. Against AGEN-AODV, SHARP-AODV delivers up to 12.1% PDR advantage, 40.4% throughput superiority, 83.4% latency improvement, and 85.5% jitter reduction, validating that AAV-specific optimizations—altitude-aware forwarding, velocity–distance channel estimation, and sliding-window buffer monitoring—yield measurable benefits beyond general-purpose GA-LA adaptive mechanisms designed for ground-based MANETs. These quantitative results empirically validate the efficacy of domain-tailored multi-parameter adaptive routing and demonstrate that intelligent AAV-specific protocols significantly enhance network performance in challenging scenarios with high-speed three-dimensional mobility, severe Doppler effects, fast topology dynamics, and heterogeneous node capabilities.

Besides overcoming the legacy routing solutions’ scaling limits, SHARP-AODV unveils broad opportunities for deployment in next-generation technology demanding stringent Quality-of-Service guarantees. With the fast-changing Metaverse environment, SHARP-AODV allows AAVs to be mobile nodes of infrastructure, facilitating real-time generation of Digital-Twin, AR/VR content streaming with ultra-low latency (<100 ms) and stable jitter (<20 ms), and an enormous-scale user immersion experience even in regions with no fixed infrastructure, thus extending advanced services to remote or disaster sites [21]. Within fog and edge computing paradigms, SHARP-AODV allows AAVs to function as agile edge nodes whose adaptive response to node density and channel conditions facilitates mobile-edge computing, distributed fog-as-a-Service architectures, and edge intelligence applications, with high PDR and low delay supporting federated AI workloads where AAV swarms collaboratively execute distributed learning and inference in real time [22,23,24]. For the emerging 6G architecture envisioned for ubiquitous connectivity, SHARP-AODV satisfies critical next-generation requirements, including URLLC for mission-critical applications such as emergency services and remote surgery, aligns with mMTC objectives targeting device densities up to 10^7^/km^2^, and seamless operation in heterogeneous space–air–ground–sea integrated networks where the multi-criteria path-selector can be co-optimized with forthcoming Terahertz communication technologies [25,26].

In smart-city and intelligent-transport contexts, SHARP-AODV-equipped AAV fleets form resilient mobile sensor networks enabling timely traffic monitoring, enhanced V2X communication channels, and accelerated emergency response capabilities that remain operational when natural disasters disrupt ground infrastructure. Following the developing technology trends and established research requirements, promising future directions include incorporating AI/ML techniques for adaptive protocol parameter optimization and mobility forecast [27], creating energy-aware adaptations that explicitly model battery consumption and charging, employing blockchain technologies to improve security and trust in distributed AAV routing choices, expanding the protocol to provide differentiated QoS for Metaverse traffic classes with heterogeneous characteristics [8], and studying cross-layer optimization mechanisms that bind routing choices to MAC-layer scheduling and physical-layer beamforming in dense deployments of AAVs.

## Figures and Tables

**Table 1:** Evolution of AAV applications across mobile-network generations.

Network Generation	Throughput	Latency	Connection Density	Key AAV Applications
4G (LTE)	<100 Mbps	~50 ms	$10^{5}$ devices/km^2^	Video recording, photography, and basic monitoring
5G	<10 Gbps	<5 ms	$10^{6}$ devices/km^2^	Cargo delivery, real-time monitoring, and HD live streaming
6G	<1 Tbps	<100 µs	$10^{7}$ devices/km^2^	AAV swarms, Metaverse AR/VR, and Digital Twin

**Table 2:** General simulation parameters.

Parameter	Value
Routing Protocol	AODV/SHARP-AODV
Wi-Fi Standard	IEEE 802.11g [20]
Propagation Model	LogDistance
Packet Size	512 bytes
Simulation Time	300 s
Simulation runs	20 per configuration
Simulation area	800 m × 800 m × 100 m
AAV Transmission Range	250 m
Traffic Type	Constant Bit Rate (CBR)
Data rate	1024 kbps
Transmission power	18 dBm
Reception threshold	−85 dBm

## Data Availability

The original contributions presented in this study are included in the article. Further inquiries can be directed to the corresponding author.
